# Efficacy of adjuvant trastuzumab in women with HER2-positive T1a or bN0M0 breast cancer: a population-based cohort study

**DOI:** 10.1038/s41598-022-05209-8

**Published:** 2022-01-20

**Authors:** Sanji Ali, Jace Hendry, Duc Le, Prosanta K. Mondal, Amer Sami, Haji Chalchal, Kamal Haider, Osama Ahmed, Ali El-Gayed, Philip Wright, Mehrnoosh Pauls, Kate Johnson, Shahid Ahmed

**Affiliations:** 1 College of Medicine, University of Saskatchewan, Saskatoon, Canada; 2 Saskatoon Cancer Center, Saskatchewan Cancer Agency, University of Saskatchewan, 20 Campus Drive, Saskatoon, SK S7N4H4 Canada; 3 Clinical Research Support Unit, University of Saskatchewan, Saskatoon, Canada; 4 Allan Blair Cancer Center, Saskatchewan Cancer Agency, Saskatoon, Canada

**Keywords:** Cancer, Breast cancer

## Abstract

Adjuvant trastuzumab has been associated with superior survival in women with ≥ T1c or node-positive HER2-positive early-stage breast cancer; however, there is a lack of phase III trials in women with T1a/bN0 disease. Our study aimed to assess the outcomes of women with HER2-positive T1a/bN0 breast cancer who received adjuvant trastuzumab in Saskatchewan, Canada. We evaluated all women diagnosed with HER2-positive T1a/bN0 breast cancer in Saskatchewan between 2008 and 2017. We performed Cox proportional multivariable analysis to determine factors correlated with survival. In addition, inverse probability treatment weighting (IPTW) using propensity score was performed to assess benefit of adjuvant trastuzumab. Ninety-one eligible women with a median age of 61 years (range 30–89) were identified. Thirty-nine (43%) women received adjuvant trastuzumab. Women who received trastuzumab were younger and had a higher rate of T1b disease. Overall, 3% of women who received trastuzumab compared to 12% of women who did not receive trastuzumab developed breast cancer recurrence (p = 0.23). Five-year disease-free survival (DFS) of women who received adjuvant trastuzumab was 94.8% compared to 82.7% of women who did not receive trastuzumab (p = 0.22). Five-year overall survival was 100% of women who received trastuzumab compared to 90.4% of women who did not receive adjuvant trastuzumab (p = 0.038). In the multivariable analysis, grade III tumors were correlated with inferior DFS (hazard ratio [HR] 5.5, 95% CI [1.7–17.7]). The propensity score using the inverse probability of treatment weighting showed that lack of adjuvant trastuzumab was correlated inferior DFS, with an HR of 4 (95% CI 1.05–15.5). Women with HER2-positive T1a/bN0 breast cancer had overall low recurrence of breast cancer. However, the results of this exploratory analysis indicate that women who received adjuvant trastuzumab had better survival.

## Introduction

HER2-targeted therapy is one of the greatest success in breast cancer research. Women with HER2-positve breast cancers have significantly better outcomes when treated with HER2 directed therapies^1,2^. Trastuzumab, a monoclonal antibody is the first HER2-targeted agent that binds the extracellular domain of the HER2 receptor^2^.

Trastuzumab initially evaluated in women with HER2-positive advanced breast cancer and showed better outcomes^3^. It was subsequently assessed in women with early-stage breast cancer. Based on the results of several randomized adjuvant trials in women with node-positive tumors or > 1 cm high-risk node-negative breast cancer, 1 year of adjuvant trastuzumab became a new standard of care for women with HER2-positive early stage breast cancer^4–6^. A 2012 Cochrane meta-analysis of eight randomized trials, involving almost 12,000 patients, which compared chemotherapy plus trastuzumab to chemotherapy alone favored the trastuzumab-containing regimens and showed better overall survival (hazard ratio [HR] 0.66, p < 0.00001) and disease-free survival (HR 0.60, p < 0.00001)^7^.

All phase III trials that evaluated the benefit of adjuvant trastuzumab, excluded women with T1a or b tumors and node-negative disease. Historically, women with T1a or bN0M0 early-stage breast cancer were considered to have a good prognosis; however, recent studies suggest that women with HER2-positive T1a or bN0M0 cancers may be at higher risk for recurrence^8–11^. A phase II APT (adjuvant paclitaxel and trastuzumab) trial assessed the benefit of adjuvant trastuzumab plus weekly paclitaxel in 406 women with T1N0 or T2N0 HER2-positive tumors measuring up to 3 cm with predominantly stage I breast cancer. Adjuvant paclitaxel plus trastuzumab was associated with a risk of early recurrence of about 2%^12^. Currently, the rate of use of adjuvant trastuzumab and the factors correlated with adjuvant trastuzumab in women with T1a or b node-negative disease are not well known.

In the absence of level I evidence, this study attempted to compare the outcomes of women with HER2-positive T1a or b node-negative disease who received adjuvant trastuzumab to those who did not receive adjuvant trastuzumab, as well as to identify the factors correlated with recurrence. Furthermore, this study aimed to determine the rate of use of adjuvant trastuzumab in patients with HER2-positive T1a or b node-negative disease and the factors correlated with adjuvant trastuzumab.

## Methods

### Study population

The research protocol was approved by the Research Ethics Committee of the University of Saskatchewan. For data access, operational approval was obtained from the Saskatchewan Cancer Agency. This was a retrospective population-based cohort study. The study population included adult women with histologically documented HER2-positive T1a/bN0M0 breast cancer, diagnosed between January 2008 and December 2017 in the province of Saskatchewan, Canada. Patients with equivocal HER2 status were excluded from the study. Additionally, patients with a secondary active malignancy or de novo metastatic breast cancer were also excluded. Eligible patients were identified using the Saskatchewan Cancer Registry. The electronic medical records of patients were individually reviewed. An abstraction sheet was used to guide data collection. Eligible patients were then divided into two subcohorts based on whether or not they received adjuvant trastuzumab.

### Statistical analysis

The demographics and baseline characteristics of the study population were translated into categorical and continuous variables. Categorical variables were compared using the chi-square test. Continuous variables were analyzed using Student’s *t*-test. Overall survival (OS) was defined as the time from the diagnosis of early-stage operable breast cancer to death from any cause. Disease-free survival (DFS) was defined as the time from the diagnosis of early-stage operable breast cancer to the date of relapse, secondary cancer or death from any cause. Invasive disease-free survival was defined as the time from the diagnosis of early-stage operable breast cancer to the date of relapse of invasive breast cancer or a new invasive primary breast cancer. Survival of the entire cohort and subgroups was estimated using the Kaplan–Meier method. The survival distribution of different groups was compared using the log rank test. The overall significance level was set at 0.05.

### Cox proportional hazard model

A multivariable Cox proportional hazard multiple regression analysis was performed to assess the correlation between adjuvant trastuzumab use and the outcomes of patients with HER2-positive T1a/bN0M0 breast cancer. In addition, various other clinical and pathological variables were examined to determine whether they were correlated with recurrence. The HR and its 95% confidence interval (95% CI) were estimated by Wald method. The likelihood-ratio test and *t*-test were used to determine whether the addition of independent variables of interest significantly improved the prediction of survival in the model^13^. The following variables were examined for their prognostic significance: age (< 50 vs. ≥ 50 years), major comorbid illness, performance status (ECOG 0 vs. > 0), smoking status, estrogen and progesterone receptor status (positive vs. negative), tumor size as a continuous variable, T status (T1a vs. T1b), tumor grade (I/II vs. III), margin, type of surgery (lumpectomy vs. mastectomy), adjuvant endocrine therapy, and adjuvant radiation. Variables with p value of less than 26 were examined in a multivariable model. 

In addition, propensity scores were calculated to reduce the effects of potential confounding because of the distribution of baseline characteristics between treatment and control groups. A logistic regression model was used to estimate propensity scores by examining various clinical and pathological variables described above. To assess the treatment effect of trastuzumab on disease-free survival, the propensity score was incorporated in the Cox model using the inverse probability of treatment weighting (IPTW) method^14^. All statistical analyses were performed using SAS 9.4 (SAS Institute, Inc., Cary, NC, USA) and SPSS (version 27, IBM, Armonk, NY, USA).

### Conference presentation

Part of the data was presented at the European Society of Medical Oncology (ESMO) Breast Cancer 2021 Congress.

### Ethical approval

All procedures performed in studies involving human participants were in accordance with the ethical standards of the institutional and/or national research committee and with the 1964 Helsinki declaration and its later amendments or comparable ethical standards. The Biomedical Research Ethic Committee of the University of Saskatchewan approved the study and waived informed consent (Bio-1634).

## Results

**Figure 1:**
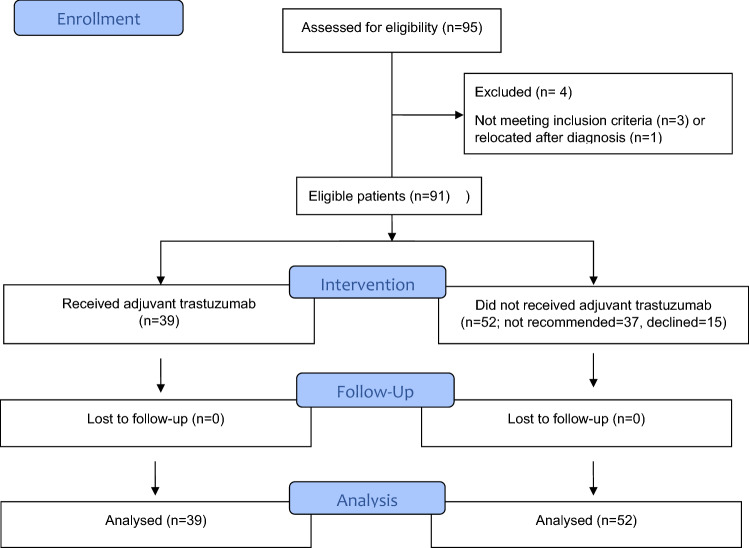
Flow diagram of eligible patients with HER2-positive T1a/b node negative breast cancer.

We identified 95 patients with HER2-positive T1a/b node-negative breast cancer and no metastatic disease (Fig. 1). Three of these patients were excluded due to a large tumor size or node-positive disease. One patient was excluded due to relocation. The median age of the study population was 61 years (range 30–89 years), and 63% had a T1b tumor. Overall, 39 (43%) women who received adjuvant trastuzumab in combination with chemotherapy were in the treatment group, and 52 (57%) women were in the control group. Patient characteristics and study variables are presented in Table 1. Both groups were comparable, although women who received trastuzumab were significantly younger than those in the control group (median age 57 vs. 65 years, p = 0.02). In addition, 92% of women who received adjuvant trastuzumab had T1b disease compared to 40% of women in the control group, and the mean tumor size was 7.8 ± 2.0 and 5.3 ± 2.6 mm, respectively (p < 0.0001). All women in the trastuzumab group received chemotherapy, while only one woman received chemotherapy in the control group. It should be noted that adjuvant chemotherapy in combination with trastuzumab was not recommended to 37 (41%) patients. For 15 (17%) patients, trastuzumab was offered by the oncologist as a preferred adjuvant therapy, but was declined by the patient. Combination of docetaxel and cyclophosphamide (DC) was the most common regimen and was administered in 30 (77%) women followed by weekly paclitaxel in  4 (10%) cases. Out of the 39 women who received adjuvant trastuzumab, 33 (85%) completed 1 year of treatment. Overall, 10 (26%) patients presented a transient decline in left ventricle ejection fraction, but no patients developed symptomatic heart failure (Table 2).

**Table 1:** Baseline characteristics of the entire study population and subgroups of patients who received adjuvant trastuzumab and those who did not receive adjuvant trastuzumab.

Variables	All patients N = 91 (%)	Treatment group N = 39 (%)	Control N = 52 (%)	p value
Age (years)	61 (range 30–89)	57 (30–76)	65 (30–89)	0.02
Comorbid illness	44 (48)	18 (46)	26 (50)	0.83
History of a secondary cancer	16 (18)	6 (15)	10 (19)	0.78
**ECOG performance status**				
0	77 (85)	31 (80)	46 (89)	0.25
1	14 (15)	9 (20)	6 (11)	
Smoking history	41 (45)	20 (50)	21 (40)	0.39
Current smoker	8 (9)	5 (13)	3 (6)	0.28
**Surgery**				
Lumpectomy	43 (47)	17 (44)	26 (50)	0.67
Mastectomy	37 (41)	14 (36)	23 (44)	0.51
Bilateral mastectomy	11 (21)	8 (21)	3 (6)	0.04
**Node exam**				
Sentinel node	77 (85)	34 (87)	43 (83)	0.77
Complete nodal dissection	13 (14)	5 (13)	8 (15)	0.77
Size in mm (mean ±)	6.4 ± 2.7	7.8 ± 2.0	5.3 ± 2.6	< 0.0001
**T status**				
Tmic	1 (1)	0	1 (2)	
T1a	33 (36)	3 (8)	30 (58)	< 0.0001
T1b	57 (63)	36 (92)	21 (40)	< 0.0001
**Grade**^a^				
I	14 (15)	6 (15)	8 (15)	1.0
II	42 (46)	14 (36)	28 (54)	0.13
III	34 (37)	18 (46)	16 (31)	0.18
Margin within 1 mm	8 (9)	3 (8)	5 (10)	1.0
**Hormone receptor status**				
ER positive	60 (66)	27 (69)	33 (64)	0.65
PR positive	45 (50)	20 (51)	25 (48)	0.83
Adjuvant systemic therapy	65 (71)	39 (100)	26 (50)	< 0.0001
Adjuvant chemotherapy	40 (44)	39 (100)	1 (2)	< 0.0001
Adjuvant endocrine therapy	53 (58)	27 (69)	26 (50)	0.08
Adjuvant radiation therapy	52 (57)	26 (67)	26 (50)	0.13

^a^Grade was unknown in one patient in the treatment group. *ECOG* Eastern Cooperative Oncology Group.

**Table 2:** Type of adjuvant chemotherapy and treatment related adverse effects in women who received adjuvant trastuzumab.

Interventions/toxicity	N = 39
**Received chemotherapy**	
Docetaxel plus cyclophosphamide	30 (77)
Weekly paclitaxel × 12	4 (10)
5FU, epirubicin, and cyclophosphamide followed by docetaxel (FEC-DOC)	4 (10)
Doxorubicin plus cyclophosphamide followed by weekly paclitaxel × 12	1 (3)
Completed all planned cycles of chemotherapy	34^a^ (87)
Febrile neutropenia	4 (10)
Completed one year of trastuzumab (17 or 18 treatments)	33 (85)
Asymptomatic drop in left ventricle ejection fraction	10 (26)
Symptomatic heart failure	0

^a^5 patients stopped chemotherapy early due to treatment related side-effects.

### Outcomes

The median follow-up period for all patients was 70 months (inter-quartile range [IQR] 48–96 months), with 68 months (IQR 49–94) in the treatment group and 73 months (IQR 46–98) in the control group. Overall, seven (8%) women developed breast cancer recurrence, with one (3%) in the trastuzumab group and six (12%) in the control group (p = 0.23; Table 3). All but one were locoregional recurrences. One patient with local recurrence subsequently developed distant recurrence.

**Table 3:** Outcomes of the entire study population and subgroups of patients who received adjuvant trastuzumab and those who did not receive adjuvant trastuzumab.

Variables	All patients N = 91 (%)	Treatment group N = 39 (%)	Control N = 52 (%)	p value
Recurrence	12 (13)	3 (10)	9 (17)	0.22
Biopsy proven	12	3	9	
**Type of recurrence**				
Local	6 (7)	1 (3)	5 (10)	0.23
Distant	1 (2)	0	1 (2)	1.0
New primary cancer	5 (6)	2 (6)	3 (6)	1.0
Breast cancer recurrence	7 (8)	1 (3)	6 (12)	0.23

**Figure 2:**
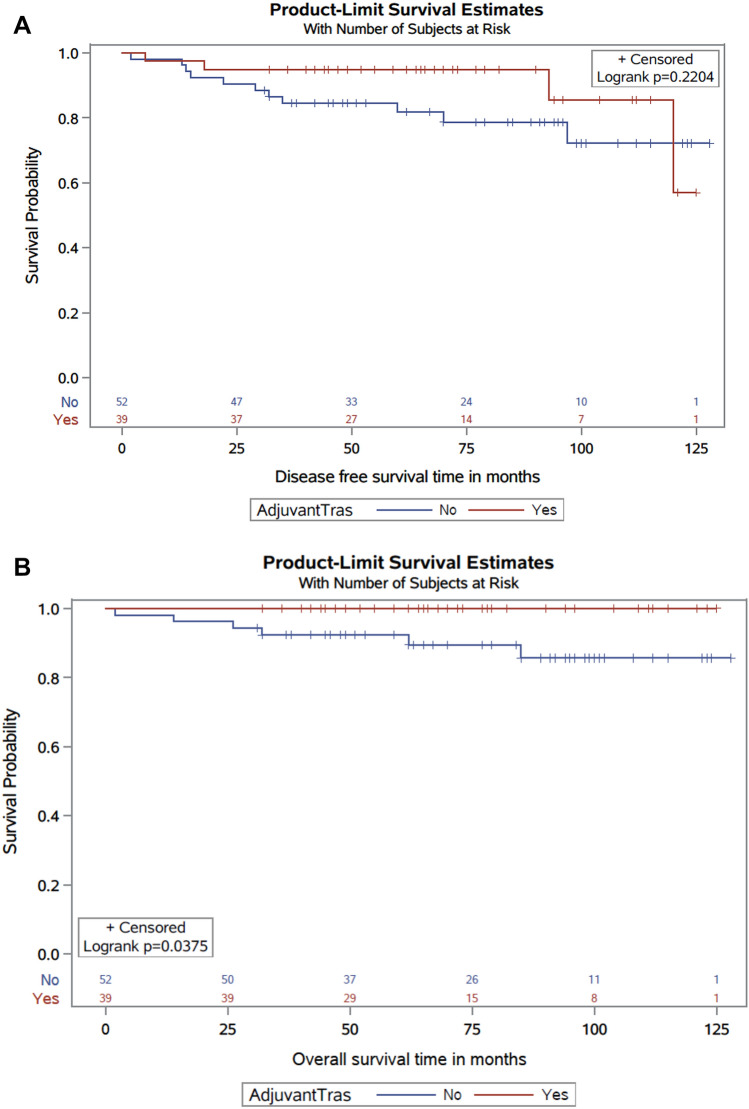
(**A**) Kaplan–Meier disease free survival curves of the adjuvant trastuzumab and control groups. (**B**) Kaplan–Meier overall survival curves of patients in adjuvant trastuzumab and control groups.

Median DFS was not reached. However, the estimated 5-year DFS was 94.8% in the trastuzumab group compared to 82.7% in the control group (p = 0.22; Fig. 2A). Five-year invasive breast cancer-free survival was 97.4% in the trastuzumab group and 94.2% in the control group (p = 0.29). Five-year overall survival was 90.4% in the control group versus 100% in women who received adjuvant trastuzumab (p = 0.038; Fig. 2B). In the multivariable analysis, grade III tumors were correlated with inferior DFS, with an HR of 5.5 (95% CI 1.70–17.7). The association between the lack of adjuvant trastuzumab and DFS was not significant (HR 3.0, 95% CI 0.92–9.5) (Table 4). The IPTW propensity score approach found that lack of adjuvant trastuzumab was associated with poorer DFS, with an HR for disease recurrence of 4 (95% CI 1.05–15.5).

**Table 4:** Cox Proportional univariate and multivariate analyses of factors associated with disease free survival in patients with T1a/bN0 HER2 + breast cancer.

Variables	HR (95% CI)	HR (95% CI)
	Univariate analysis	Multivariate analysis
Age < 50	1.3 (0.30–5.9)	…
Comorbid illness	1.0 (0.36–2.8)	…
ECOG performance status 1	1.02 (0.23–4.6)	…
Current smoking	1.7 (0.37–7.8)	…
Hormone receptor positive	1.9 (0.53–6.7)	…
Size	1.02 (0.85–1.2)	…
T status	1.4 (0.48–4.2)	…
Not received adjuvant trastuzumab	2.0 (0.64–6.3), p = 0.23	3.0 (0.92–9.5)
Grade 3	4.9 (1.56–15.5), p = 0.007	5.5 (1.70–17.7)
Positive margin	1.8 (0.39–7.9)	…
Not received Adjuvant endocrine therapy	1.06 (0.37–3.0)	…
Not received adjuvant radiation	1.5 (0.50–4.6)	…
Mastectomy	2.4 (0.76–7.6), p = 0.13	1.9 (0.60–6.2)
HR using IPTW method		
Not received adjuvant trastuzumab		4 (1.05–15.5)

*CI* confidence interval, *HR* hazard ratio, *IPTW* inverse probability of treatment weighting.

## Discussion

The results of this study demonstrate that patients with small (T1a/b) HER2-positive node-negative (N0) breast cancer have a low risk of recurrence. Five-year invasive breast cancer-free survival was about 97% in patients who received adjuvant trastuzumab compared to 94% in the control group. In addition, late recurrence was higher in the control group, as 97% of patients in the trastuzumab group were free from breast cancer recurrence at 10 years compared to 88% of patients in the control group. However, this difference did not reach statistical significance, possibly due to the relatively small number of events in the study cohort. Most recurrences were locoregional recurrences. Despite the low risk of recurrence in both groups, the study findings suggest that adjuvant trastuzumab further reduces the risk of recurrence, as evidenced by the lower number of recurrences in the treatment group.

Patients in the trastuzumab group had a better 5-year DFS compared to the control group, with a difference between the treatment and control groups of about 12%. Furthermore, the study demonstrated better OS in the adjuvant trastuzumab group. In the control group, two patients died from recurrent advanced breast cancer, one due to a second, advanced primary cancer and one from an unrelated cause.

In the multivariable analysis, the risk of a DFS event in patients with a high-grade tumor was five-fold higher than in those with lower-grade tumors. Tumor grade is known to be a prognostic variable in breast cancer. Other studies have also shown that high-grade early-stage breast cancer with a tumor size of 1 cm or smaller is associated with poorer DFS^15,16^. An analysis using the propensity score showed that a lack of adjuvant trastuzumab was associated with a fourfold higher risk of a DFS event. Propensity score is a balancing score. IPTW reduces or eliminates systematic differences between the observational and treatment groups to a comparable degree and allows a nonrandomized study to mimic a randomized controlled trial^14^.

Using the Netherlands Cancer Registry, Van Ramshorst et al. retrospectively examined the benefit of adjuvant trastuzumab in a large cohort of women with T1 tumors up to 2 cm. The majority of women received it in combination with chemotherapy. Overall, 45% of patients received trastuzumab. Adjuvant trastuzumab was associated with a significant improvement in 8-year OS of 95% vs. 84% with observation alone^17^. A small French study assessed the benefit of adjuvant trastuzumab and found no recurrence in women who received adjuvant treatment compared with 2 (10.5%) recurrences in women who did not receive adjuvant therapy^18^.

A recent systemic review and meta-analysis of seven studies involving 1181 patients with T1a or bN0 HER2-positive breast cancer revealed that approximately 47% of patients receive adjuvant trastuzumab^19^. The pooled analysis showed that patients who received adjuvant trastuzumab had a lower risk of recurrence compared to those who did not (odds ratio 0.201, 95% CI [0.100, 0.404], p < 0.001). Of note, in our patient cohort, other variables including tumor size, age and hormone receptor status were not correlated with DFS. Due to the small number of recurrence and survival events, multivariable analyses for invasive breast cancer-free survival and OS were not performed.

Overall, 43% patients in our study cohort received adjuvant trastuzumab, which is similar to the rate reported in other studies^19^. All patients received adjuvant chemotherapy. Age and tumor size were found to be correlated with adjuvant trastuzumab use. Women in the adjuvant trastuzumab group were younger than women in the control group (median 57 vs. 65 years, respectively). In addition, 92% of women who received trastuzumab had T1b tumors. Hence, a younger age and larger tumor size (T1b) were associated with the use of adjuvant trastuzumab. Although 26% of patients experienced a drop in left ventricle ejection fraction, all cases were transient and no patients developed symptomatic heart failure. No other patients developed any major complications related to trastuzumab. Overall, 85% of patients completed 1 year of adjuvant trastuzumab treatment.

Although one year of adjuvant trastuzumab is the standard duration of treatment, there is some evidence that 6 months of adjuvant trastuzumab is non-inferior to one year of treatment and is associated with a lower rate of cardiotoxicity^20,21^. For example, the PERSEPHONE trial demonstrated that four-year DFS in patients who received 6 months of trastuzumab was non-inferior to those who received 12 months of trastuzumab (89.4% vs. 89.8%)^20^. Overall, patients with node negative T1a or Tb HER2 positive breast cancer would derive a smaller benefit with HER2 directed therapy compared to patients with node positive or larger HER2 positive breast cancer. Hence, treatment toxicity is an important consideration when contemplating adjuvant HER2 directed therapy in smaller node-negative tumors. The small absolute difference in the outcomes of women who were treated with 1 year vs. a shorter duration suggests that 6 months of trastuzumab is a reasonable option in women with low risk HER2 positive small tumors.

Anthracycline or platinum based chemotherapy, such as a combination of doxorubicin, cyclophosphamide and paclitaxel (ACT) or docetaxel and carboplatin (TC), respectively, are the common adjuvant chemotherapy backbone that are used in combination with trastuzumab, with or without pertuzumab, in patients with high risk HER2 positive breast cancer^22^. Both regimens are associated with high risk short term and long-term side effects. The optimal chemotherapy regimen for a node negative small HER2 positive breast cancer is not known, but the single agent paclitaxel in combination with trastuzumab is an appropriate option that has a low rate of serious adverse effects^12^. In our cohort, the majority of patients received a combination of docetaxel and cyclophosphamide as a primary chemotherapy backbone with trastuzumab. However, after publication of the phase II APT trial, weekly single agent paclitaxel was also used as a common regimen in combination with trastuzumab. A recent randomized phase II ATTEMPT trial compared the combination of trastuzumab and weekly paclitaxel to antibody–drug conjugate trastuzumab emtansine (T-DM1) in women with stage I HER2 positive breast cancer. The rate of clinically relevant toxicity was similar between the 2 groups (47% vs. 46%)^23^. It is not known if a non-chemotherapy HER2 directed treatment can be used without compromising the outcome of patients with small node negative HER2 positive breast cancer. Dual HER2 blockade therapies without chemotherapy have demonstrated efficacy in the preoperative setting in patients with HER2-positive breast cancer^24^. However, data about non-chemotherapy HER2 blockade including single agent trastuzumab is limited in the adjuvant setting^25^. Future trials are warranted to explore this option in smaller HER positive breast cancer.

Our findings suggest that adjuvant trastuzumab confers some benefit in patients with early-stage breast cancer with a smaller tumor size and node-negative disease. The results of this study highlight the need for future phase III clinical trials to further investigate the role of adjuvant trastuzumab along with a non-chemotherapy dual HER2 blockade to minimize toxicity in early-stage T1a and T1b node-negative HER2-positive breast cancer. However, given the small number of events in women with T1a or T1b tumors and the fact that adjuvant trastuzumab is being used in such women in clinical practice, it is not feasible to complete a randomized trial with observation alone as the control in this group of women.

It is important to highlight the limitations of the current study, which include the small sample size and the retrospective nature of the study. In addition, as it was a retrospective study, beside febrile neutropenia we did not collect information on chemotherapy-related toxicity. However, one of the key strengths of this population-based study is that all patients who were diagnosed with T1a or T1b HER2-positive breast cancer in the province of Saskatchewan were evaluated, and there was no selection bias or loss to follow-up. In addition, instead of using administrative data, individual medical records were reviewed to ensure the accuracy of the information.

In summary, this retrospective non-randomized study showed that younger women and those with T1a/bN0 disease tend to receive adjuvant trastuzumab and that adjuvant trastuzumab was associated with lower recurrence and better overall survival.

## Supplementary Information

Supplementary Information.
