# Supplementary material for: Efficacy of adjuvant trastuzumab in women with HER2-positive T1a or bN0M0 breast cancer: a population-based cohort study
Source: Sci Rep. 2022 Jan 20;12:1068. doi: 10.1038/s41598-022-05209-8 (PMC8776836; doi:10.1038/s41598-022-05209-8)
Supplement: Supplementary file 1 — Supplementary Information. [file 41598_2022_5209_MOESM1_ESM.docx]

**Supplement**

Inverse probability of treatment weighting (IPTW) method

For IPTW method, the followings steps were performed:

Step 1: Propensity score for each individual (same as predicted probability of getting treatment after adjusting covariates in the model) was performed using multivariable logistic regression model in relation to adjuvant trastuzumab (**Table A**).

Step 2: Weight was calculated for each individual, which is a function of propensity score and proportion of adjuvant trastuzumab.

Step 3: The calculated weight was used in the Cox regression model in relation to adjuvant trastuzumab. This model provided HR, p-value for disease-free survival for the use of adjuvant trastuzumab (**Table B**).

**Table A: Logistic regression model (propensity score model) for adjuvant trastuzumab.**

| **Effects** | **Odd ratio (95% CI)** |
| --- | --- |
| Age < 50 years | 2.35 (0.42-13.25) |
| Comorbid illness | 0.69 (0.21-2.28) |
| ECOG performance status >0 | 0.89 (0.19-4.13) |
| ERPR negative | 0.08 (0.01-0.70) |
| Size | 1.11 (0.77-1.62) |
| T1b | 19.16 (1.96-187.4) |
| Grade III | 2.45 (0.78-7.64) |
| Margin | 1.04 (0.16-6.63) |
| Adjuvant endocrine therapy | 9.32 (1.27-68.36) |
| Breast conserving surgery | 1.45 (0.45-4.68) |

**Table B: Hazard ratio of disease recurrence in relation to adjuvant trastuzumab using inverse probability of treatment weighting**

| **Intervention** | **Hazard ratio for Disease-Free Survival** |
| --- | --- |
| Not received adjuvant trastuzumab | 4 (95% CI: 1.05-15.5) |
